# Graphene and Other Nanomaterial-Based Electrochemical Aptasensors

**DOI:** 10.3390/bios2010001

**Published:** 2012-01-13

**Authors:** Frank J. Hernandez, Veli Cengiz Ozalp

**Affiliations:** 1 Department of Internal Medicine, Roy J. and Lucille A. Carver College of Medicine, University of Iowa, 375 Newton Rd, Iowa City, IA 52242, USA; 2 Institute for Polymer Materials (Polymat), University of the Basque Country, Avda. Tolosa 72, San Sebastian 20018, Spain; E-Mail: cengizozalp@gmail.com

**Keywords:** aptamers, aptasensors, biosensors, electrochemistry, nanobiotechnology, graphene, carbon nanotubes

## Abstract

Electrochemical aptasensors, which are based on the specificity of aptamer-target recognition, with electrochemical transduction for analytical purposes have received particular attention due to their high sensitivity and selectivity, simple instrumentation, as well as low production cost. Aptamers are functional nucleic acids with specific and high affinity to their targets, similar to antibodies. However, they are completely selected *in vitro* in contrast to antibodies. Due to their stability, easy chemical modifications and proneness to nanostructured device construction, aptamer-based sensors have been incorporated in a variety of applications including electrochemical sensing devices. In recent years, the performance of aptasensors has been augmented by incorporating novel nanomaterials in the preparation of better electrochemical sensors. In this review, we summarize the recent trends in the use of nanomaterials for developing electrochemical aptasensors.

## 1. Introduction

Electrochemical aptasensors (aptamer-based sensors) facilitate simple, effective and rapid detection of biomolecules which are important in medicine, environment and food applications. Aptasensors are getting considerable interest due to their unique capabilities compared to other biorecognition elements. The artificial aptamer selection procedures allow the delivery of custom-made biorecognition for any kind of target. In recent years, numerous electrochemical biosensors have been reported using a variety of nanomaterials such as quantum dots, gold nanoparticles, and carbon nanotubes. Among novel nanomaterials, carbon nanostructures provide the basis for emerging technologies in the field of molecular electronics devices. A number of recent reports specifically introduced graphene as an emerging nanomaterial for improved electrochemical sensors [1,2]. 

Carbon materials have been widely employed in analytical electrochemistry, outperforming the traditional electrodes. Their performance originates largely from structural polymorphism and chemical stability. Micro-structured carbon materials, such as carbon nanotubes (CNT), improved electrochemical applications by enabling novel designs in sensing, electrocatalysis and electronics, compared to traditional carbon materials such as glassy carbon, diamond or carbon black. Most recently, graphene emerged as a material with future potential for improved applications. These expectations are based on the more advanced properties of graphene compared to CNTs [3]. 

Graphene is a two-dimensional single atom thick nanomaterial, which has recently been at the scientific focus due to its remarkable mechanical and electronic properties. In comparison to CNTs, graphene has the advantages of high thermal and electrical conductivity due to small thickness and large surface area. Integration of graphene with metal nanoparticles is usually necessary for enhancing biosensing properties. The attachment of graphene on electrodes and biorecognition elements was achieved through a plethora of nanocomposites. Major additional premises of graphene compared to other carbon based nanomaterials are low cost and large production scale in biosensor development.

Electrochemical aptasensors integrate the aptamer functionality with the sensitivity of electrochemical technologies for detecting a variety of targets [4,5]. In the following sections we review some of the recent efforts to construct novel and improved electrochemical sensors involving graphene, carbon nanotubes and some metal nanoparticle composites after short introductory summaries on aptamers and amperometric, potentiometric or impedimetric measurements. 

## 2. Aptamers

Aptamers are target affinity nucleic acids selected artificially via a combinatorial procedure. They have frequently been proposed as alternatives to antibodies with additional desirable properties. In the last decades, due to a variety of materials with unique properties for sensor development, aptasensors have been given more attention for developing novel analytical tools [6,7,8,9,10]. The *in vitro* selection procedure, SELEX (systematic evolution of ligands by exponential enrichment), is a powerful technique which can identify functional nucleic acid sequences with specific affinity to desired target molecules [11,12]. The selection methodologies can be designed to deliver aptamers with specific affinity strengths for the desired targets [13,14]. Moreover, post-SELEX procedures can improve affinity of aptamers based on the initial selection [15,16,17]. Physical persistence is another useful characteristic of aptamers compared to antibodies; extreme pH or temperature treatments do not interfere with nucleic acid refolding ability, whereas most antibodies are completely destroyed under such conditions. Therefore, aptamers have sensitivity and selectivity comparable to antibodies, and additionally they have extended shelf life [18]. 

Interactions of nucleic acids with various nanomaterials allowed improved designs of biosensors [19]. For example, physical adsorption of aptamers on carbon-based materials resulted in numerous aptasensors, this simplifying the manufacturing procedures of many optical or electrochemical sensors [20,21,22]. Similarly, straightforward procedures for stable strong attachment of aptamers to nanomaterials allowed the development of sensitive and reusable aptasensors based on fluorescence [23], colorimetry [24], luminescence [25], quartz crystal microbalance (QCM) [26], interferometry [27], plasmon resonance [28] or localized surface plasmon resonance [29] and electrochemistry. The most common surface functionalizations include covalent, gold-thiol, or biotin-streptavidin attachments. Four basic strategies were employed during the fabrication of aptasensors [30]; (i) target-induced structure switching or (ii) displacement, (iii) sandwich, and (iv) competitive modes. 

## 3. Amperometric, Potentiometric and Impedimetric Aptasensors

### 3.1. Amperometric Measurements

**Figure 1:**
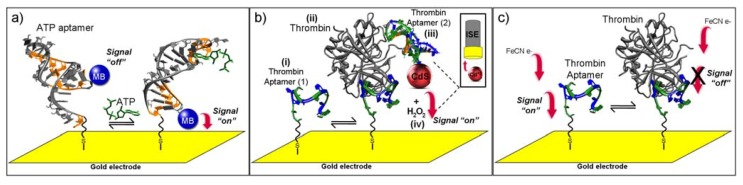
Typical aptasensor strategies for: (**a**) amperometric, (**b**) potentiometric or (**c**) impedimetric measurements. In panel (a), ATP binding aptamer labeled with methylene blue (MB) at one end was immobilized on gold surface at the other end. Specific interaction between aptamer sequence and ATP induces a conformational change, which significantly changes the electron transfer distance between MB and the gold surface. In panel (b), a primary thrombin binding aptamer is immobilized on gold surface via a sulfhydryl bond (i). In the presence of thrombin (ii), the secondary thrombin aptamer (CdS-modified) binds the complex (iii). After addition of H_2_O_2_ (iv), the Cd^2+^ ions are detected by the ion-selected electrode (ISE). In panel (c), thrombin binding aptamer (PDB: 1QDF) is immobilized on gold surface as in (b) and the interaction between aptamer and thrombin (PDB: 1HAO) can be monitored by impedimetric measurements in the presence of ferricyanide.

Amperometric sensors determine the presence of target by detecting the changes in current resulting from the electrochemical oxidation or reduction of an electroactive species. It is usually performed by maintaining a constant potential at working electrode with respect to a reference electrode. The resulting current is directly correlated to the bulk concentration of the electroactive compound. This signal-transduction mechanism is frequently used for enzymatic and catalytic biosensors. The main advantage of this class of transducer is the low cost, therefore disposable electrodes are often used with this technique. The high degree of reproducibility that is possible for these (one time use) electrodes eliminates the cumbersome requirement for repeated calibration. The type of instrument used for these measurements is also very easy to obtain and can be inexpensive and compact, this allowing for the possibility of *in situ* measurements. Limitations for this signal transduction mechanism include the potential interferences to the response, if several electroactive compounds generate false current values. These effects have been eliminated for clinical applications through the use of selective membranes, which carefully control the molecular weight or the charge of compounds that have access to the electrode. Figure 1(a) shows a scheme of an amperometric aptasensor for the detection of ATP.

### 3.2. Potentiometric Measurements

Potentiometric sensors measure the potential difference between a working and a reference electrode or two reference electrodes separated by a selective membrane, when there is no significant current flowing between them. The transducer is usually an ion-selective electrode (ISE). The main advantage of such devices is the wide concentration range for which ions can be detected, generally between 10^−6^ to 10^−1^ mol/L. Their continuous measurement capability is also an interesting advantage for many applications. The apparatus is inexpensive, portable, and it is well suited for *in situ* measurements. The main disadvantage is that the limit of detection for some samples can be high (10^−5^ mol/L or 1 ppm) and the selectivity can be poor. One of the attractive characteristics of potentiometric measurements with ISE is the relative independence of signal from sample volume [31]. 

One reported application of potentiometric aptasensors demonstrated the usefulness of ion-selective design in a microsensor for detecting biomolecular interactions [32]. Thrombin molecules were captured by aptamers immobilized on a gold surface while a second thrombin aptamer labeled with CdS nanoparticles was allowed to interact with captured thrombin molecules. The captured cadmium was dissolved by peroxide treatment and the potentiometric response was measurement via a polymer membrane Cd^2+^ ion selective microelectrode (Figure 1(b)). More examples of potentiometric aptasensors can be found in a recent review by Sassolas *et al*. [33].

### 3.3. Conductimetric/Impedimetric Measurements

Electrochemical impedance spectroscopy (EIS) measures the response (current and its phase) of an electrochemical system to an applied oscillating potential as a function of the frequency. EIS is a rapidly developing electrochemical technique for the investigation of bulk and interfacial electrical properties of any kind of solid or liquid material which is connected to an appropriate electrochemical transducer. Moreover, the EIS method is label-free, simple, and requires no external modification of biomolecules. In the field of biosensors, it is particularly well-suited to the detection of binding events on the transducer surface. Figure 1(c) shows the detection of thrombin binding on immobilized aptamers by EIS measurements.

The large applicability of conductimetric detection is due to the observation that almost all enzymatic reactions involve either consumption or production of charged species. The electric field is generated using a sinusoidal voltage (AC) which helps in minimizing undesirable effects such as Faradic processes, double layer charging and concentration polarization. The primary advantage of this technique is the use of inexpensive, reproducible and disposable sensors. The main disadvantage is that the ionic species produced must significantly change the total ionic strength to obtain a reliable measurement. This requirement increases the detection limit to unacceptable levels and results in potential interferences from variability in the ionic strength of the sample. High sensitivity of the method is highly advantageous, but also can be associated with nonspecific impedance changes that could be easily mistaken for specific interactions. 

Aptamers have been used in conductimetric/impedimetric sensors in a variety of designs in order to exploit direct biomolecule measurements [33,34]. Examples involving aptamers associated with graphene, carbon nanotubes and various nanoparticles will be discussed in detail in the appropriate sections below. 

### 3.4. Field-Effect Transistors

Sensors based on field effect transistors (FET) rely on electrical response modulation of conductors. FET consists of a source, a drain and a gate. The current in gate voltage can be regulated by the voltage between source and drain. The gate terminal can be modified with a molecular receptor or ion-selective membrane for establishing a biosensor. The binding of target analyte on molecular receptors is detected as an electrical signal based on the dependence of gate voltage and channel conductance [35]. Aptamers have been used as the molecular recognition element in a variety of FET-based biosensors. An aptamer-based carbon nanotube FET was reported for immunoglobulin E (IgE) [36] and thrombin quantification [37]. More recently, graphene FET aptasensors have been developed by Ohno and co-workers for immunoglobulin G (IgG) [38] and IgE [39]. Other nanomaterials used in FET-based aptasensors include polymer nanotubes [40], silica nanowires [41], poly(3,4-ethylenedioxythiophene) nanowires [42], and polypyrol nanotubes [43].

## 4. Graphene-Based Nanocomposites

Graphene is an excellent conductor material and thus, graphene modified electrodes exhibit fine electrochemical response [2]. The outstanding properties of graphene created extensive interests among researchers, whereas how to fully exploit the unique properties to fabricate novel graphene-nanocomposites-based devices remains a challenge. To this end, a variety of approaches have been reported for electrochemical biosensors development. Surface area constitutes an essential characteristic for biosensing, biocatalysis and energy storage applications [3]. Graphene is reported to have a wide electrochemical potential window (2.5 V in PBS buffer), with an AC impedance spectra showing a low charge-transfer resistance. Detailed reviews of electrochemical properties of graphene can be found elsewhere [44,45,46,47]. 

The strong interaction between graphene and single stranded nucleic acids is another advantage that has been used to develop simple and effective electrochemical aptasensors. Covalent modifications on the graphene surfaces have also been exploited for aptamer immobilization. Researchers have usually combined graphene with various nanomaterials in order to facilitate development of electrochemical sensors.

**Figure 2:**
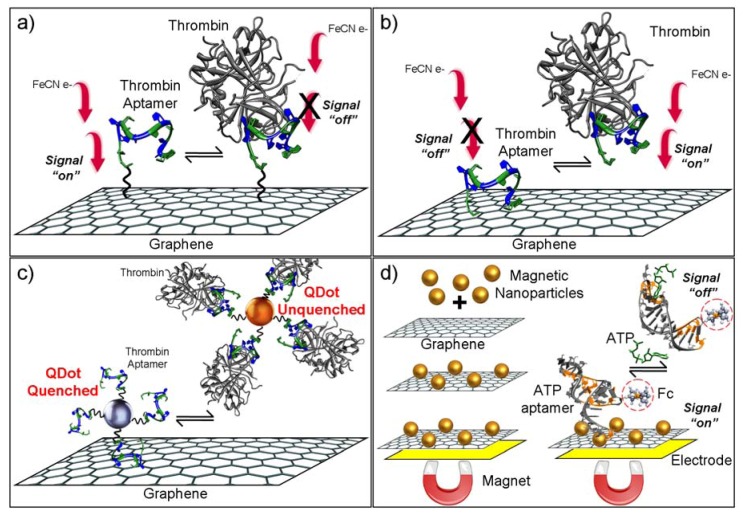
Approaches for preparing graphene-based electrochemical sensing platforms. An impedimetric sensor with (**a**) covalent and (**b**) non-covalent aptamer immobilization, (**c**) graphene quantum dots, (**d**) magnetic nanoparticles-graphene bioelectronics.

Aptamers can be immobilized on graphene nanocomposites via covalent bonds for developing reusable sensors or via physical adsorption of aptamers on graphene to obtain one time use sensors. Both strategies have been demonstrated to be useful in aptasensor development. 3,4,9,10-perylene tetracarboxylic acid (PTCA) is an archetypal π-stacking organic perylene dye with favorable photo and chemical stability. PTCA strongly adsorbs on graphene through π-π stacking and thus prevents graphene aggregation. Another advantage of PTCA composites is the added carboxyl groups which can be exploited for covalent attachment of aptamers. Graphene-promoted PTCA (GPD) nanocomposites has been synthesized as redox probe for developing an electrochemical thrombin aptasensor [48]. The authors reported a novel redox sensor by achieving a well-defined cathodic peak which was not observed previously with graphene. A detection range of 0.001 nM to 40 nM with limit of detection at 200 fM was obtained for thrombin detection (Figure 2(a)). In a similar application of PTCA-graphene nanocomposite, an electrochemical aptasensor was developed for detection of cancer cells by using nucleolin binding (AS1411) aptamer [49]. Nucleolin is a marker protein for cancer cells which is overexpressed on the tumor cell membranes. EIS measurements were employed to detect binding of cancer cells on the electrode surface with a detection limit of 794 cells/mL. The reported detection limit and dynamic range were better compared to a chemiluminescent sensor [50] and a nanofiber based electrochemical sensor [51] with the same aptamer sequence. Detection limit of this graphene based sensor was comparable to those of previously reported single-walled carbon nanotube (SWCNT) based aptasensor (620 cells/mL) [52]. However, graphene based cytosensor can be considered an improvement due to a lower production cost. The constructed graphene nanocomposite sensor surface was also tested with MTT (Methylthiazolyldiphenyl-tetrazolium bromide) assay for cytotoxicity and found to be non-toxic.

Structure-switching of aptamers has been fruitful in designing electrochemical biosensors. A sensitive aptasensor was reported based on gold nanoparticles-graphene nanosheets using structure-switching histidine aptamers [53]. Gold nanoparticles were deposited on graphene through spontaneous chemical reduction of chloroauric acid by sodium citrate and then the complex of nanoparticle-graphene was immobilized on glassy carbon electrodes. Subsequently, ferrocene modified aptamers were immobilized on the gold surface of the complex via gold-thiol bonds. The aptamer consisted of ferrocene modified form of a catalytic DNA strand which hybridized to aptamer sequence. Thus, the binding event of histidine on the aptamer sequence results in degradation of single stranded DNA fragment and ferrocene approaches to the surface by increasing electrical signal. This specific and sensitive sensor achieved 0.1 pM detection limit.

A couple of graphene based formats were reported by using aptamer adsorption on the graphene and then electrochemical signal was recorded by target induced release of the aptamer molecules [54]. Wang and co-workers described an electrode by well-dispersed graphene oxide on gold films by chemical reduction of hydrazine. The target-induced aptamer release was used to detect the amount of thrombin by surface plasmon resonance and impedance spectroscopy (Figure 2(b)). Zhao and co-workers employed ultrafine graphene quantum dots (GQD) for the fabrication of a thrombin electrochemical sensor [55]. The GQD modified electrode was readily prepared by following a straightforward incubation procedure of graphene with pyrolytic graphite electrode (Figure 2(c)). Differential pulse voltammetry measurements were used for the detection of single stranded complementary DNA and the aptamer ligand, thrombin. 

A small molecule electrochemical sensor was designed by using target-induced release of ferrocene-labeled aptamers from a magneto-controlled graphene surface [56]. In magneto-controlled bioelectronics approach, an external magnetic field is used to achieve electrical contact between a nanocomposite (graphene and magnetic nanoparticles) and the electrode [57]. Magnetic graphene nanosheets (MGP) can be prepared by following a facile procedure by attaching iron oxide nanoparticles to graphane sheets. Magnetic field is an efficient way to attach MGP on gold surface. Ferrocene-labeled aptamers attach to MGP due to non-covalent attraction between nucleobase and grapheme, resulting in a strong electrochemical signal. Adenosine 5'-triphosphate (ATP) specifically interacted with aptamers immobilized on the nanosheet surface, displacing the aptamers from the surface electrode, thus inducing a decrease in electrochemical signal observed (Figure 2(d)). The method was further validated for small molecule detection by using a different aptamer (cocaine binding aptamer). 

Finally a complex of silica-graphene-gold based system was developed as ATP sensor. Guo and co-workers reported a triplex-DNA design of electrochemical aptasensor by using silver microspheres (SMS) as separation element and graphene-mesoporous silica-gold nanoparticle nanocomposite (GSGH) as sensing platform [58]. In this design, ATP binding aptamers were immobilized on SMS through a thiol bond and a complementary DNA was hybridized to the aptamer sequence. ATP binding releases complementary DNA sequence into solution where it is captured by a complementary DNA immobilized on the electrodes. The presence of captured probe decreases the electrical signal from ferrocene labeled poly(ethyleneimide) (PEI) deposition. Therefore, GSGH platform provides a surface with reduced unspecific adsorption of biological materials and good immobilization of complementary probes on gold surfaces. 

## 5. Carbon Nanotubes

Carbon nanotubes (CNT) and their hybrids have attracted great interest in biosensor development due to their unique properties. Large surface to volume ratio is the major characteristic of CNTs. Nucleic acids can readily be adsorbed on the surfaces of carbon nanotubes through the π-π stacking. Various sensing formats have been created by using displacement of adsorbed aptamers from the nanotube surfaces in the presence of complementary DNA, aptamer target, exonucleases or some dye molecules such as methylene blue. Reusable sensors have been designed through covalent attachment of aptamers to hybrid components of graphene containing composites. Two groups of CNTs can be synthesized; single-walled carbon nanotubes (SWCNT) or multi-walled carbon nanotubes (MWCNT). SWCNTs are formed when a graphene sheet is rolled to a tube and two or more concentric tubes form MWCNTs. Detailed information on synthesis of carbon nanotubes and their electrochemical applications can be found in some recent reviews [59,60,61,62,63]. 

SWCNTs have abundant accessible surface area for biological immobilization and their high electron mobility, low electrical resistance, favorable biocompatibility make them excellent candidates for biosensing. They can promote the electron transfer and improve the catalytic activity of nanoparticles. Bai and co-workers functionalized platinum-gold nanoparticles and horseradish peroxidase with SWCNTs to label thrombin aptamer for developing an ultrasensitive electrochemical biosensor [64]. An aptamer displacement assay was developed for small molecules by forming a conduction channel composed of SWCNTs between the source and drain electrodes. A capture DNA fragment was covalently immobilized on the SWCNT surfaces and aptamers were immobilized by hybridization. Using displacement principle upon target binding, the sensor detected as low as 1 pM ATP [65]. An ultrasensitive thrombin aptasensor was reported to have a limit of detection of 8.3 fM by dual amplification strategy [66].

Kara and co-workers developed a thrombin aptasensor based on impedance spectroscopy. MWCNTs were attached on the surface of screen-printed carbon electro-transducers. Aptamers were attached on the carbon nanotubes through amine bond and the thrombin binding was monitored by resistance to charge transfer for obtaining a limit of detection of 105 pM. In a similar report, MWCNTs were modified with thrombin aptamers hybridized to ferrocene labeled complementary DNA [67]. Target induced release of ferrocene labeled DNA fragment out of the electrode surface was monitored as a significant decrease in the measurements of differential pulse voltammetry (DPV). In another variation of the similar approach, MWCNTs were attached on glassy carbon electrode surface, thrombin aptamers were adsorbed on nanotubes through non-covalent attachment, and target induced aptamer release was detected by EIS measurements [68]. Tran and co-workers (2011) developed an amperometric sensor by polymerizing polyaniline-multiwalled carbon nanotube film on interdigitated platinum electrode arrays for the detection of human papillomavirus (HPV) infection [69]. They immobilized peptide aptamers HPV-16-L1 as affinity capture for the antigen peptide aptamer HPV-16-L1 (1,825 Da) and its specific antibody HPV-16 (150 kDa). Due to the larger size of the antibody compared to the immobilized aptamer, the binding of the aptamer to the complex antigen/antibody results in a change of electroactivity that can be detected in a direct, label-free format.

## 6. Metal Nanoparticle-Based Strategies

Tris(2,2'-bipirydine)ruthenium(II) (Ru(bpy)_3_^2+^) based platforms have been known for high electrochemical stability and reversible behavior. An ultrasensitive thrombin aptasensor was designed based on solid state Ru(bpy)_3_^2+^ ECL [70]. A composite of ruthenium and platinum nanoparticles was immobilized on the surface of nafion coated glassy carbon electrodes. The aptamers were attached to the electrode surface by a streptavidin-biotin bond. In a similar approach, sensitive detection of thrombin was reported via poly(pyrrole-*co*-pyrrole propylic acid) nanoparticles loaded with aptamer and ruthenium complex [71]. The nanoparticles were synthesized by an alcohol-assisted microemulsion polymerization. The sensor was fabricated by covalent coupling of nanoparticles on the surface of paraffin-impregnated graphite electrode. This study reported a detection limit of 3 × 10^−16^ M.

Li and co-workers developed a novel biosensor by immobilizing thrombin binding aptamer molecular beacon on soluble CdSe quantum dots modified on top of the glassy carbon electrode [72]. Methylene blue was intercalated into aptamer molecules as a means of electrochemical labeling. The authors reported on the detection of thrombin by differential pulse voltammetry (DPV). The use of CdSe quantum dots improved electrochemical signal mostly because of their larger surface area.

Another interesting study described an electrochemical aptasensor, based on disposable screen-printed electrodes for the detection of the mycotoxin ochratoxin A (OTA) [73]. Two strategies were investigated by using an indirect and a direct competitive assay, based on the use of superparamagnetic nanoparticles. The best strategy was found to be the direct competitive format. In this assay, free OTA competed with labeled alkaline phosphatase (ALP)-OTA for the binding of the DNA aptamer immobilized on magnetic beads. The electrochemical detection was achieved by differential pulse voltammetry for the enzymatic reaction of ALP and its substrate.

To improve the sensitivity of an impedimetric sensor, 15 nm gold nanoparticles (AuNP) bearing thrombin-binding aptamers were immobilized on the surface of gold electrodes via hexanedithiol [74]. The transduction principle is based on electron transfer resistance in the presence of an [Fe(CN)6]^3^^−^/^4^^−^ redox couple, which can be measured by electrochemical impedance spectroscopy. The linear range of detection for the sensor was demonstrated to be 1–30 nM thrombin.

## 7. Future Prospects

The exceptional properties of carbon-based nanomaterials make them compelling for electrochemical biosensor development. Aptamers have already proven to be one of the potentially best suited biorecognition elements in nanomaterial-based sensing platforms. The complex nanocomposites consisting of carbon nanomaterials, various nanoparticles and aptamers promise label-free, ultrasensitive biosensors at reasonable costs for any analyte of interest. The graphene-nanoparticle combinations provide improved electrochemical platforms and aptamer incorporation serves as a unique element in achieving a universal biosensor for any desired target. Recently reported applications, as proof-of-principle studies, have been confined to a couple of aptamer sequences (mostly thrombin or ATP binding aptamers). Future trends in electrochemical aptasensor research should broaden the spectrum of applications in medical diagnosis, environmental monitoring and other biosensing fields.
